# Bi-handed assembly chaperones regulate protein complex assembly through an intramolecular handover mechanism

**DOI:** 10.1126/sciadv.adw9158

**Published:** 2025-09-12

**Authors:** Jingyi Wu, Chun Wan, Yuan Tian, Yan Ouyang, Harrison Puscher, Suzhao Li, Qian Yin, Jingshi Shen

**Affiliations:** ^1^ Department of Molecular, Cellular and Developmental Biology, University of Colorado, Boulder, CO 80309, USA.; ^2^ Department of Biological Sciences and Institute of Molecular Biophysics, Florida State University, Tallahassee, FL 32306, USA.; ^3^ Department of Medicine, University of Colorado Anschutz Medical Campus, Aurora, CO 80045, USA.

## Abstract

A critical yet challenging step in protein complex assembly is the formation of a dimeric intermediate that serves as a seed for incorporating additional subunits. We hypothesized that this step could be facilitated by “bi-handed” chaperones that recognize two different subunits through distinct domains (hands). However, whether such chaperones exist remained unknown. Here, we identify AAGAB as a bona fide bi-handed chaperone. AAGAB uses its C-terminal domain (CTD) to bind the α subunit and its GTPase-like domain (GD) to bind the σ2 subunit of the AP2 adaptor complex, a central player in membrane trafficking. AAGAB first recruits α via its CTD; σ2 then joins through interaction with α, forming a conformationally immature α:σ2 hemicomplex at the CTD. This hemicomplex is subsequently transferred to the GD via a GD:σ2 binding interface, accompanied by conformational maturation. These findings establish AAGAB as the founding member of a bi-handed chaperone family and reveal an intramolecular handover mechanism that underlies their mode of action.

## Teaser

A bi-handed assembly chaperone is found, and an intramolecular handover mechanism is revealed in protein complex assembly.

## INTRODUCTION

Approximately half of eukaryotic proteins function within protein complexes (*1*, *2*). Folding chaperones, such as Hsp70s and chaperonins, assist the folding of individual protein subunits, but these subunits attain their native three-dimensional conformations only within the fully assembled protein complex (*1*, *3*–*6*). Individual subunits and assembly intermediates expose hydrophobic interfaces and are susceptible to aggregation, misassembly, and degradation (*1*, *3*, *4*, *7*, *8*). Moreover, macromolecular crowding in the cellular milieu decreases the diffusion rates of individual protein subunits, interferes with their proper association, and favors misassembly (*9*–*11*). Thus, while protein complexes may form spontaneously in vitro or in heterologous expression systems (*12*–*14*), their assembly in the native cellular environment is assisted by assembly chaperones (also known as assisting factors or assembly factors) (*3*, *5*, *7*, *15*–*17*). Assembly chaperones stabilize individual subunits and assembly intermediates, facilitate their proper interactions, and dissociate upon the formation of full protein complexes (*3*, *5*, *18*–*22*). Unlike the well-characterized folding chaperones, the functions and mechanisms of assembly chaperones remain poorly understood.

Despite their vast diversity in composition and size, protein complexes generally begin their assembly processes with the pairing of two separate subunits into a dimeric intermediate complex, which then serves as a “seed” for the incorporation of additional subunits (*3*, *23*, *24*). Because individual protein subunits are inherently unstable and short-lived in their free, soluble form, it is highly inefficient for them to assemble correctly through random encounters. A similar challenge arises in chemical reactions involving two reactants, where enzymes overcome this limitation by bringing substrates into close proximity, a principle known as the proximity effect (*25*). By positioning two substrates within the enzyme’s active site, their collision frequency increases, thereby accelerating the chemical reaction (*25*). Thus, it is conceivable that if an assembly chaperone has two substrate-recognizing domains (or “hands”), it could take advantage of the proximity effect to bring two protein subunits together to form a dimeric complex. However, it remains unknown whether such bi-handed assembly chaperones exist.

In this work, we found that AAGAB, a poorly characterized protein that assists the assembly of the AP2 adaptor complex (*17*, *26*, *27*), is a bona fide bi-handed assembly chaperone. The AP2 adaptor is a heterotetramer composed of two large subunits (α and β2), one medium subunit (μ2), and one small subunit (σ2) (*28*–*32*). AP2 assembles in the cytosol before being targeted to the plasma membrane, where it captures cargo and recruits clathrin to promote clathrin-mediated endocytosis (CME) (*12*, *33*–*38*). We found that AAGAB binds to the σ2 and α subunits of AP2 through its guanosine triphosphatase–like domain (GD) and C-terminal domain (CTD), respectively, and facilitates their pairing into a dimeric α:σ2 hemicomplex. The AAGAB-assisted assembly of the α:σ2 hemicomplex is an unexpectedly complex process involving sequential molecular interactions. When α and σ2 are recruited to AAGAB, an α:σ2 hemicomplex first forms at the CTD of AAGAB, but this hemicomplex remains conformationally immature. Subsequently, the α:σ2 hemicomplex is transferred from the CTD to the GD of AAGAB, accompanied by its conformational maturation. Together, these studies uncover the founding member of a bi-handed assembly chaperone family and establish an intramolecular handover mechanism for the chaperone.

## RESULTS

### AAGAB has two substrate-binding domains

**Fig. 1.**
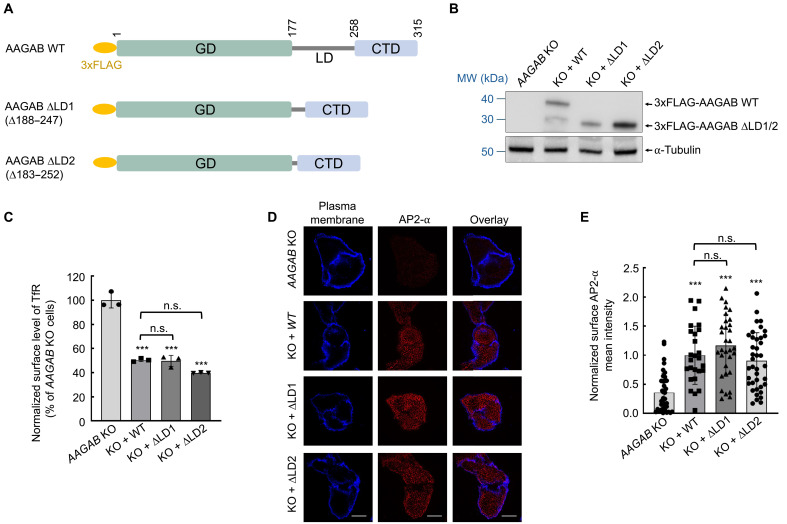
The LD is dispensable for the function of AAGAB in AP2 assembly. (**A**) Diagrams of WT and mutant AAGAB proteins. (**B**) Representative immunoblots showing the expression of the indicated proteins in HeLa cells. (**C**) Flow cytometry measurements showing normalized surface levels of TfR in the indicated HeLa cell lines. Data normalization was performed by setting the mean value of *AAGAB* KO cells to 100%, and all data points, including the *AAGAB* KO samples, were normalized to that mean value. Approximately 5000 cells were measured for each sample. Data are presented as mean ± SD of three biological replicates. ***P < 0.001 (compared to *AAGAB* KO cells); not significant (n.s.) *P* > 0.05, calculated using one-way analysis of variance (ANOVA) with Holm-Sidak corrections. (**D**) Representative confocal microscopy images showing AP2 puncta (α staining) on the plasma membrane in the indicated HeLa cell lines. The plasma membrane was stained with CF405-conjugated concanavalin A. Images were captured using a 100× oil immersion objective on a Yokogawa/Olympus CV1000 spinning disk confocal microscope. Scale bars, 10 μm. (**E**) Quantification of AP2 puncta on the plasma membrane. Images were captured as in (D) and analyzed using ImageJ. Each dot represents imaging data from an individual cell. Data of all samples were normalized to those of *AAGAB* KO cells expressing the WT rescue gene. Error bars indicate SD. ***P < 0.001 (compared to *AAGAB* KO cells); n.s. *P* > 0.05, calculated using one-way ANOVA with Holm-Sidak corrections. MW, molecular weight.

Alignment of human AAGAB and its orthologs in other species revealed two conserved domains: an N-terminal GD (residues 1 to 177 in human AAGAB) and a CTD (residues 258 to 315 in human AAGAB) (Fig. 1A and fig. S1A) (*26*, *39*). In biochemical assays using recombinant AAGAB and AP2 subunits coexpressed in *Escherichia coli*, the CTD of AAGAB directly bound the α subunit of AP2 (fig. S1, B to E) (*26*), while the GD interacted directly with the σ2 subunit (fig. S1, B to E). In the absence of AAGAB coexpression, soluble α and σ2 could not be obtained in *E. coli* (fig. S1, B to E) (*17*, *26*). These data demonstrate that AAGAB contains two separate substrate-recognizing domains that directly bind the α and σ2 subunits of AP2.

Next, we examined whether there is another substrate-binding domain in AAGAB. The GD and CTD of AAGAB are connected by a linker domain (LD; residues 178 to 257), which is less conserved and predicted to be largely unstructured (fig. S1A) (*26*). To determine whether the LD is required for the function of AAGAB, we deleted most of the LD sequence and examined whether the LD mutant (D188-247, ΔLD1) is still functional in the cell (Fig. 1, A and B). Surface levels of the transferrin receptor (TfR), an AP2-dependent CME cargo (*17*, *26*, *27*), are elevated in *AAGAB* knockout (KO) human cells (Fig. 1C). We observed that surface TfR levels were similarly restored when either wild-type (WT) AAGAB or the LD deletion mutant was expressed in *AAGAB* KO cells (Fig. 1C). We also generated another LD deletion mutant (D183-252, ΔLD2), which removed nearly the entire LD (Fig. 1, A and B). This LD mutant also restored surface TfR levels to a similar degree as WT AAGAB (Fig. 1C). When expressed in *AAGAB* KO human cells, both of the LD deletion mutants rescued mature AP2 adaptors on the plasma membrane (Fig. 1, D and E). Thus, the LD is dispensable for the function of AAGAB in the cell, indicating that the GD and CTD constitute a minimal complement in regulating AP2 assembly. This finding agrees with the observation that AAGAB does not bind to the β2 or μ2 subunit (*17*, *40*). These results demonstrate that AAGAB uses its GD and CTD to recruit two separate AP2 subunits, and there is no third substrate-binding domain in AAGAB, suggesting that AAGAB is a bi-handed assembly chaperone.

### The GD and CTD of AAGAB must be physically linked in regulating AP2 assembly

**Fig. 2.**
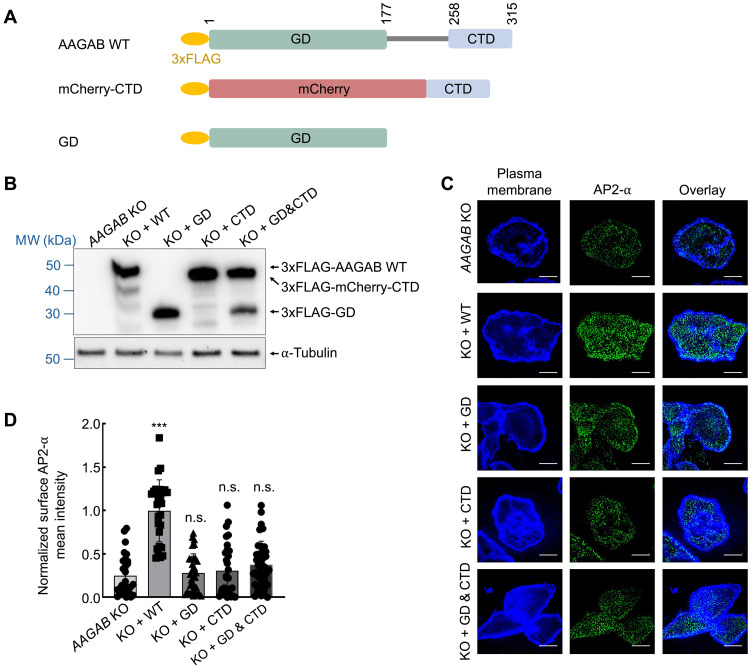
The GD and CTD of AAGAB must be physically linked in regulating AP2 assembly. (**A**) Diagrams of WT and mutant AAGAB proteins. (**B**) Representative immunoblots showing the expression of the indicated proteins in HeLa cells. (**C**) Representative confocal microscopy images showing AP2 puncta (α staining) on the plasma membrane of the indicated HeLa cell lines. Images were captured using a 100× oil immersion objective on a Yokogawa/Olympus CV1000 spinning disk confocal microscope. Scale bars, 10 μm. (**D**) Quantification of AP2 puncta on the plasma membrane. Images were captured as in (C) and analyzed using ImageJ. Each dot represents imaging data from an individual cell. Data of all samples were normalized to those of *AAGAB* KO cells expressing the WT rescue gene. Error bars indicate SD. ***P < 0.001; n.s. *P* > 0.05 (compared to *AAGAB* KO cells, calculated using one-way ANOVA with Holm-Sidak corrections).

We reason that, if AAGAB is a bona fide bi-handed chaperone, its two substrate-binding domains must be physically linked to achieve the proximity effect. To test this prediction, we severed the AAGAB protein into the GD and CTD fragments (Fig. 2A) and examined whether coexpression of the two fragments could regulate AP2 assembly in human cells. Since short peptides are often unstable in the cell (*41*), an mCherry tag was added to the N terminus of the CTD to stabilize it (Fig. 2A). Our previous work showed that N-terminal tagging does not affect AAGAB’s activity (*17*, *26*, *27*). The GD and CTD fragments were expressed in *AAGAB* KO human cells, both individually and in combination (Fig. 2B). We found that expression of either the GD or CTD alone in *AAGAB* KO cells failed to restore mature AP2 adaptors on the cell surface (Fig. 2, C and D), confirming that the function of AAGAB requires both the GD and CTD. Coexpression of the GD and CTD in *AAGAB* KO cells did not restore surface AP2 adaptors either (Fig. 2, C and D). These results demonstrate that the GD and CTD of AAGAB must be physically connected to regulate AP2 assembly, further supporting our conclusion that AAGAB is a bona fide bi-handed assembly chaperone relying on the proximity effect to regulate substrate pairing.

### The two substrate-binding domains of AAGAB are spatially flexible in regulating AP2 assembly

**Fig. 3.**
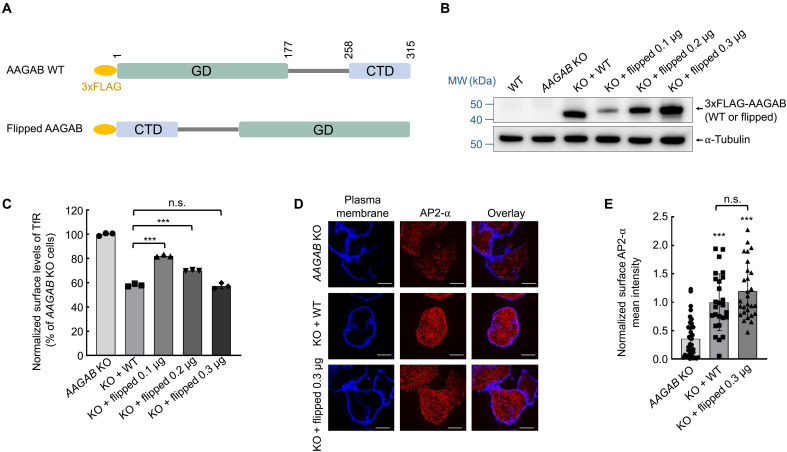
A flipped AAGAB variant is functional in regulating AP2 assembly. (**A**) Diagrams of WT AAGAB and the flipped AAGAB variant (amino acids 258 to 315, 178 to 257, and 1 to 177). (**B**) Representative immunoblots showing the expression of the indicated proteins in *AAGAB* KO HeLa cells. Cells grown on a 24-well plate were transfected with the indicated amounts of plasmids (0.1 to 0.3 μg for each well). (**C**) Flow cytometry measurements showing normalized surface levels of TfR in the indicated HeLa cell lines. Data are presented as mean ± SD of three biological replicates. ***P < 0.001; n.s. *P* > 0.05, calculated using one-way ANOVA with Holm-Sidak corrections. (**D**) Representative confocal microscopy images showing AP2 puncta (α staining) on the plasma membrane of the indicated HeLa cell lines. Images were captured using a 100× oil immersion objective on a Yokogawa/Olympus CV1000 spinning disk confocal microscope. Scale bars, 10 μm. (**E**) Quantification of AP2 puncta on the plasma membrane. Images were captured as in (D) and analyzed using ImageJ. Each dot represents imaging data from an individual cell. Data of all samples were normalized to those of *AAGAB* KO cells expressing the WT rescue gene. Error bars indicate SD. ***P < 0.001 (compared to *AAGAB* KO cells); n.s. *P* > 0.05, calculated using one-way ANOVA with Holm-Sidak corrections.

In enzyme-catalyzed chemical reactions, many enzymes not only hold substrates close together but also place them into an optimal orientation to accelerate the reaction (*25*). Next, we sought to determine whether this orientation effect is also involved in the chaperone function of AAGAB. To this end, we generated a flipped AAGAB variant, switching the GD and CTD domains to disrupt their spatial orientation (Fig. 3A). The flipped AAGAB variant was expressed in *AAGAB* KO human cells at various levels (Fig. 3B). We observed that the flipped AAGAB variant rescued surface TfR levels in a dose-dependent manner (Fig. 3C). When WT AAGAB and the flipped variant were expressed at comparable levels, surface TfR was similarly restored (Fig. 3, B and C). The flipped AAGAB variant also fully restored endogenous mature AP2 adaptors on the cell surface when expressed in *AAGAB* KO cells (Fig. 3, D and E). Consistent with these findings, coimmunoprecipitation (co-IP) showed that the flipped AAGAB variant bound normally to α and σ2 in human cells (fig. S2). Thus, the position of the GD and CTD in AAGAB is flexible and does not encode spatial information demanded by an orientation effect, consistent with our observation that the LD can be deleted without affecting the function of AAGAB (Fig. 1). These results indicate that AAGAB’s chaperone function in AP2 assembly depends on the proximity effect but not the orientation effect.

### Formation of an α:σ2 hemicomplex at the CTD of AAGAB

**Fig. 4.**
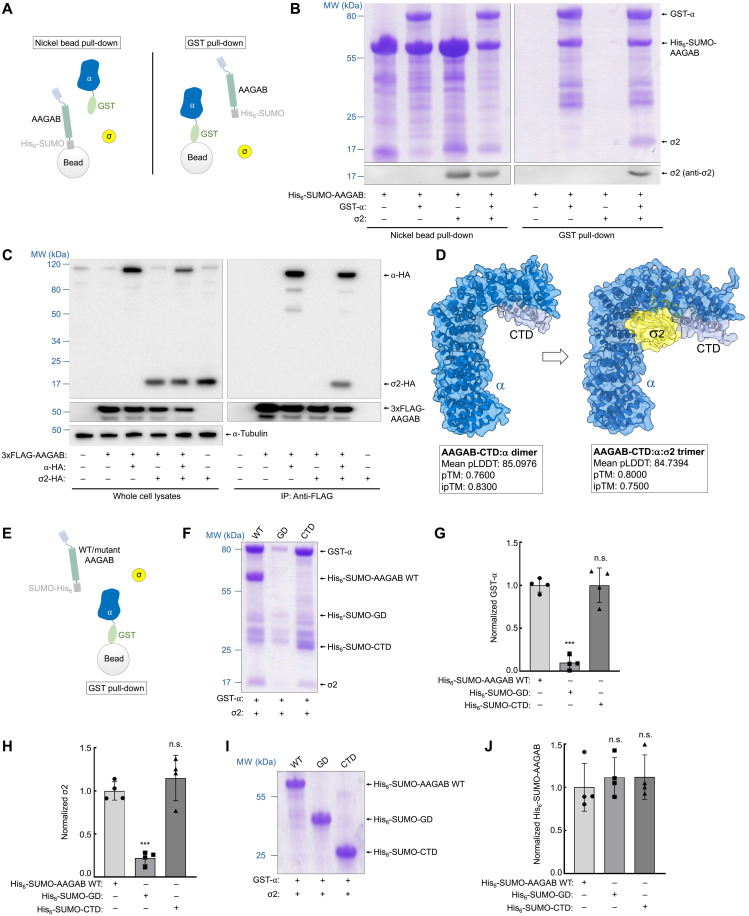
Formation of an α:σ2 hemicomplex at the CTD of AAGAB. (**A**) Diagram of pull-down assays detecting AAGAB binding to α and σ2. His_6_-SUMO–tagged WT AAGAB was coexpressed with GST-tagged α (trunk domain, amino acids 1 to 621), untagged WT σ2, or both in *E. coli*. Lysates from *E. coli* expressing the indicated proteins were used for pull-down assays. (**B**) Representative Coomassie blue–stained gels (top) and immunoblots (bottom) showing the binding of His_6_-SUMO–tagged WT AAGAB to GST-tagged α, untagged σ2, or both, as depicted in (A). (**C**) Representative immunoblots showing the interaction of 3xFLAG-tagged AAGAB with HA-tagged α and σ2 in AAGAB KO HeLa cells. AAGAB was immunoprecipitated using anti-FLAG antibodies, and proteins in the immunoprecipitates were detected via immunoblotting. (**D**) AlphaFold-predicted structural models of the AAGAB-CTD:α dimer and the AAGAB-CTD:α:σ2 trimer. The dimer prediction was performed using the CTD (amino acids 258 to 315) of AAGAB and the α trunk domain, whereas the trimer prediction was performed using the CTD of AAGAB, the α trunk domain, and WT σ2. The PDB/CIF files of these structural models are included in datasets S1 and S3. (**E**) Diagram of a GST pull-down assay detecting the binding of AAGAB (WT, GD, or CTD) to α and σ2. (**F**) Representative Coomassie blue–stained gels showing the binding of AAGAB to GST-tagged α and untagged σ2 as depicted in (E). (**G** and **H**) Quantification of GST-tagged α and untagged σ2 based on Coomassie blue–stained gels from four independent experiments, including data shown in (F). Data are presented as mean ± SD. ***P < 0.001; n.s., *P* > 0.05 (calculated using one-way ANOVA with Holm-Sidak corrections). (**I**) Representative Coomassie blue–stained gels showing His_6_-SUMO–tagged AAGAB isolated using nickel beads from the samples described in (E). (**J**) Quantification of His_6_-SUMO–tagged AAGAB based on Coomassie blue–stained gels.

Next, we sought to determine the molecular mechanism by which the bi-handed chaperone AAGAB regulates AP2 assembly. When AAGAB was coexpressed with either α or σ2 in *E. coli*, dimers of AAGAB:α and AAGAB:σ2 were formed (Fig. 4, A and B), confirming that the GD and CTD can independently bind to their respective substrates (fig. S1). When coexpressed with both α and σ2 in *E. coli*, AAGAB interacted with them to form an AAGAB:α:σ2 trimer (Fig. 4B) (*17*, *26*, *40*). In human cells, AAGAB interacted with α alone as well as with σ2 in the presence of α (Fig. 4C), consistent with the results of biochemical assays using recombinant proteins (Fig. 4B and fig. S1). However, we did not detect an interaction of σ2 with WT AAGAB or the GD of AAGAB in human cells using co-IP (Fig. 4C and fig. S2) (*17*). Thus, while σ2 can directly bind to the GD of AAGAB in vitro (Fig. 4, A and B, and fig. S1), this interaction either does not occur in the native cellular environment or is too transient to detect.

To understand how AAGAB recruits α and σ2 at the molecular level, we used AlphaFold to predict the structures of AAGAB-AP2 complexes and then experimentally tested the predicted structural models. AlphaFold predicted a high-confidence structure of the AAGAB-CTD:α dimer (Fig. 4D, left, fig. S3A, and dataset S1). According to this structural model, the CTD of AAGAB interacts with an N-terminal region of α, involving the α1, α3, α5, and α7 helices (Fig. 4D and fig. S3, A and B). AAGAB binding helps shield exposed hydrophobic residues on α (fig. S3F), which are buried in the fully assembled AP2 adaptor (*34*). Three hydrophobic residues in AAGAB’s CTD—F262, F266, and L269—interact with the α5 and α7 helices of the α subunit (fig. S3B). AlphaFold also predicted a high-confidence structure of the AAGAB-GD:σ2 dimer (fig. S4A and dataset S2). By contrast, AlphaFold failed to generate high-confidence structural models using σ2 and the CTD of AAGAB as input, or using α and the GD as input, consistent with our biochemical data showing that these proteins do not form complexes (fig. S1, B to E).

Since α binds stably to AAGAB in human cells, whereas an AAGAB:σ2 interaction is not detected (Fig. 4C and fig. S2), we posit that an α:σ2 hemicomplex forms at the CTD of AAGAB. In support of this hypothesis, AlphaFold predicted a structure of a trimeric AAGAB-CTD:α:σ2 complex with an interface predicted template modeling (ipTM) score of 0.77, using α, σ2, and the CTD of AAGAB as input (Fig. 4D, right; fig. S3, C and D; and dataset S3). According to this structural model, σ2 directly interacts with the α subunit in the AAGAB-CTD:α:σ2 trimer, and there is no obvious direct contact between σ2 and the CTD of AAGAB, despite their physical proximity (Fig. 4D and fig. S3, C and D). Compared to the structural model of the AAGAB-CTD:α dimer, the CTD of AAGAB shifts upward in the CTD:α:σ2 trimer (fig. S3E). This shift distorts the α1 and α3 helices of the α subunit and disrupts CTD binding to the α5 and α7 helices observed in the AAGAB-CTD:α dimer (fig. S3E). In the AAGAB-CTD:α dimer, F262, F266, and L269 of the CTD primarily interact with the α5 and α7 helices of α (fig. S3B). In contrast, within the AAGAB-CTD:α:σ2 trimer, these CTD residues no longer contact α7 and instead predominantly interact with α3 (fig. S3D). Thus, σ2 recruitment is accompanied by a conformational change at the AAGAB-CTD:α binding interface. Together, these structural models suggest that an α:σ2 hemicomplex is formed at the CTD of AAGAB and is attached to it via a binding interface between α and the CTD of AAGAB.

Next, we examined the physiological relevance of the AlphaFold-predicted structures. The structural models of the AAGAB-AP2 complexes exhibit high-quality metrics (e.g., ipTM scores of 0.75 or higher), which are generally indicative of accurate structural predictions, with an accuracy comparable to experimental structural determination (*42*–*46*). Moreover, the molecular interactions in these structural models are mediated by evolutionarily conserved residues of AAGAB (fig. S1A), consistent with their biological importance. The validity of the structural models is also supported by a previous observation that mutations in F262, F266, and L269 of AAGAB disrupted its binding to the α subunit in vitro using recombinant proteins (*26*). To test the role of these hydrophobic residues in human cells, we used co-IP to examine how their mutations affect AAGAB:α binding. We found that mutations in F262, F266, and L269 strongly reduced the association of AAGAB with α in human cells (fig. S5), confirming the importance of these residues as predicted by AlphaFold. To further evaluate the structural models, we coexpressed WT and truncated AAGAB proteins with α and σ2 in *E. coli* (Fig. 4E). This experiment is important because the predicted local distance difference test (pLDDT) score at the AAGAB-CTD:α interface decreases upon σ2 binding (fig. S3, B and D). When coexpressed with both α and σ2, the CTD of AAGAB formed an AAGAB-CTD:α:σ2 trimeric complex (Fig. 4F), confirming the existence of this protein complex predicted by AlphaFold (Fig. 4D).

The CTD fragment was as efficient as WT AAGAB in recruiting α and σ2 and forming a trimeric complex (Fig. 4, F to H). When the GD of AAGAB was coexpressed with α and σ2 in *E. coli*, no significant amounts of the trimeric complex were formed (Fig. 4, F to H). The expression levels of the CTD and GD in *E. coli* were comparable to those of WT AAGAB (Fig. 4, I and J). These results indicate that, unlike the CTD, the GD of AAGAB cannot initiate the formation of the α:σ2 hemicomplex, in line with our model that the α:σ2 hemicomplex is formed at the CTD of AAGAB (Fig. 4D). Thus, when σ2 binds to the GD of AAGAB, the resulting AAGAB:σ2 dimer represents a dead-end configuration that cannot recruit α, providing a molecular explanation for why this binding is not observed in cells (Fig. 4C). Small amounts of α and σ2 were found bound to the GD of AAGAB when coexpressed in *E. coli* (Fig. 4, F to H), suggesting that although the GD alone cannot initiate hemicomplex formation, it is capable of binding to and stabilizing hemicomplexes that spontaneously form in *E. coli*. These findings also demonstrate that AlphaFold accurately predicts the composition and structure of AAGAB-AP2 complexes. Together, these data showed that when α and σ2 are recruited to AAGAB, an α:σ2 hemicomplex forms at the CTD of AAGAB.

### Handover of the α:σ2 hemicomplex from the CTD to the GD of AAGAB

**Fig. 5.**
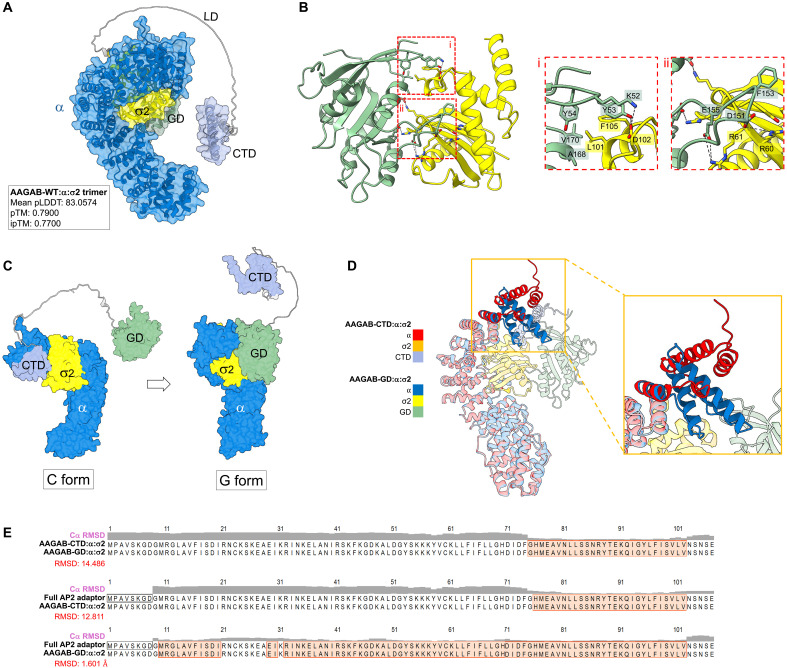
Handover of the α:σ2 hemicomplex from the CTD to the GD of AAGAB. (**A**) AlphaFold-predicted structural model of the AAGAB:α:σ2 trimer using WT AAGAB, α (the trunk domain), and WT σ2 as input. The PDB/CIF file of the predicted structure is included in dataset S4. CIF, Crystallographic Information Framework (**B**) Binding interface between σ2 and the GD of AAGAB within the AAGAB:α:σ2 trimer shown in (A). (**C**) Diagrams depicting the two forms of the AAGAB:α:σ2 trimer. The diagram of the C form is based on the AlphaFold-predicted structure of the AAGAB-CTD:α:σ2 trimer shown in Fig. 4D and the structural models of the GD and LD from the predicted structure of WT AAGAB (AlphaFold Protein Structure Database: AF-Q6PD74-F1). The LD is included in the diagrams but is dispensable for the function of AAGAB in AP2 assembly as demonstrated in Fig. 1. (**D**) Superposition of the AlphaFold-predicted structural models of the AAGAB-CTD:α:σ2 and AAGAB-GD:α:σ2 trimers. The inset shows the N terminus of the α subunit. (**E**) Structural comparison of the N terminus (amino acids 1 to 107) of the α subunit between the indicated protein complexes. The full AP2 adaptor is based on a crystal structure (PDB: 4UQI) (*34*), while other complexes are based on AlphaFold predictions. RMSD scores were calculated using UCSF ChimeraX.

While the CTD of AAGAB orchestrates the formation of the α:σ2 hemicomplex, the GD is also essential for AAGAB’s function in AP2 assembly (Fig. 2). Next, we sought to determine whether the GD plays a role in AP2 assembly beyond its dynamic binding to σ2. Using WT AAGAB, α, and σ2 as input, AlphaFold predicted a high-confidence structure of an AAGAB-WT:α:σ2 trimer (Fig. 5A, fig. S6A, and dataset S4). This structural model, predicted using WT AAGAB, is distinct from the predicted structure of the AAGAB-CTD:α:σ2 trimer (Fig. 4D, right). According to the structural model of the AAGAB-WT:α:σ2 trimer, α and σ2 also form a hemicomplex, but this hemicomplex is attached to the GD, not the CTD of AAGAB (Fig. 5, A and B). The α:σ2 hemicomplex associates with the GD through a GD:σ2 interface, and there is no direct contact between α and the GD despite their physical proximity (Fig. 5, A and B, and fig. S6A). At the GD:σ2 interface within the AAGAB:α:σ2 trimer, K52 of the GD forms a salt bridge with D102 of σ2, while Y53 forms hydrogen bonds with the same residue (Fig. 5B). Although Y54 does not directly interact with σ2, it likely plays a structural role by stabilizing the positions of K52 and Y53. D151 forms a salt bridge with R60 of σ2, and E155 forms salt bridges with R61 and R10. F153 inserts into a hydrophobic pocket formed by Y62 and V88 of σ2. In addition, A168 and V170 of the GD engage in hydrophobic interactions with L101 and F105 of σ2 (Fig. 5B).

A similar structural model was predicted when α, σ2, and the GD of AAGAB were used as input (fig. S6, B to D, and dataset S5). Overall, the GD:σ2 binding interface is similar in the predicted structures of the AAGAB-WT:α:σ2 trimer and the AAGAB-GD:σ2 dimer, with a root mean square deviation (RMSD) score of 0.921 Å (fig. S4B). The σ2-binding residues of AAGAB in these complexes are evolutionarily conserved (fig. S1A), supporting the biological relevance of the structural models.

The structural models described above suggest two distinct configurations of the AAGAB:α:σ2 trimer. In one configuration, referred to as the “C” form, the α:σ2 hemicomplex is bound to the CTD of AAGAB (Fig. 5C). In the other configuration, designated as the “G” form, the α:σ2 hemicomplex is associated with the GD of AAGAB (Fig. 5C). Since the α:σ2 hemicomplex is initially formed at the CTD of AAGAB (Fig. 4), these analyses suggest that the α:σ2 hemicomplex is transferred from the CTD to the GD of AAGAB during AP2 assembly (Fig. 5C). In support of this model, AlphaFold predicted the G form of the trimer using WT AAGAB as input (Fig. 5, A and B), suggesting that the G form reflects a lower energy state downstream of the C form.

To understand why the α:σ2 hemicomplex is transferred from the CTD to the GD of AAGAB, we compared the structural models of the AAGAB-CTD:α:σ2 and AAGAB-GD:α:σ2 trimers with the crystal structure of the fully assembled AP2 adaptor [Protein Data Bank (PDB): 4UQI] (*34*). We observed that while σ2 adopts a similar conformation in all three structures (fig. S7), the conformation of the α subunit differs markedly (Fig. 5, D and E). In particular, an N-terminal region (residues 1 to 107) of the α subunit is markedly distorted in the C form of the trimer compared to the fully assembled AP2 adaptor (RMSD = 12.811 Å) (Fig. 5, D and E). This N-terminal region interacts with the β2 subunit in the fully assembled AP2 adaptor such that its configuration is critical for proper AP2 assembly (*34*). By contrast, the N-terminal region of α in the G form of the trimer is highly similar to that in the full AP2 adaptor (RMSD = 1.601 Å) (Fig. 5, D and E). These analyses indicate that the α:σ2 hemicomplex initially formed at the CTD of AAGAB is still conformationally immature. While this immature configuration allows for the initial pairing of α and σ2, the CTD-to-GD handover of the α:σ2 hemicomplex is necessary to achieve its conformational maturation.

Together, these observations reveal an intramolecular handover mechanism for the formation of the α:σ2 hemicomplex. The α:σ2 hemicomplex is first formed at the CTD of AAGAB, but it remains conformationally immature. Subsequently, the α:σ2 hemicomplex is transferred from the CTD to the GD of AAGAB, accompanied by its conformational maturation. Thus, both the C and G forms of the AAGAB:α:σ2 trimer represent key intermediates in the AP2 assembly pathway.

### The intramolecular handover mechanism is crucial for the proper formation of the α:σ2 hemicomplex

**Fig. 6.**
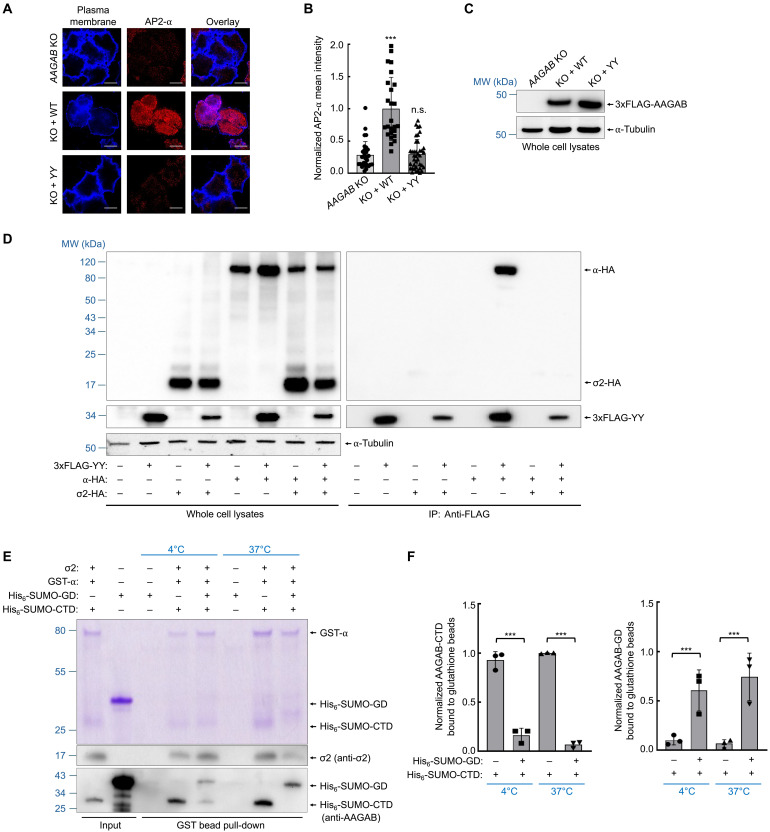
The intramolecular handover mechanism is required for proper assembly of the α:σ2 hemicomplex. (**A**) Representative confocal microscopy images showing AP2 puncta (α staining) on the plasma membrane of *AAGAB* KO HeLa cells and KO cells expressing either WT AAGAB or a YY mutant (Y53R/Y54R). Images were captured using a 100× oil immersion objective on a Yokogawa/Olympus CV1000 spinning disk confocal microscope. Scale bars, 10 μm. (**B**) Quantification of AP2 puncta on the plasma membrane. Images were captured as in (A) and analyzed using ImageJ. Each dot represents imaging data from an individual cell. Data from all samples were normalized to those of *AAGAB* KO cells with a WT rescue gene. Error bars indicate SD. ***P < 0.001; n.s. *P* > 0.05 (compared to *AAGAB* KO cells, calculated using one-way ANOVA with Holm-Sidak corrections). (**C**) Representative immunoblots showing the expression of the indicated proteins in HeLa cells. (**D**) Representative immunoblots showing the interaction of the 3xFLAG-tagged AAGAB YY mutant with HA-tagged α and σ2 in HeLa cells. (**E**) Representative Coomassie blue–stained gel showing the transfer of α and σ2 from the CTD to the GD of AAGAB. The AAGAB-CTD:α:σ2 ternary complex was isolated from *E. coli* using glutathione beads and incubated with lysates of *E. coli* expressing AAGAB-GD. After incubation for 1 hour at 4° or 37°C, the glutathione beads were washed, and proteins bound to the beads were detected using Coomassie blue staining (top) or immunoblotting (bottom). (**F**) Quantification of proteins bound to glutathione beads. Intensities of proteins were normalized to those of GST-α. Data are presented as mean ± SD of three biological replicates. ***P < 0.001 (calculated using one-way ANOVA with Holm-Sidak corrections).

Next, we experimentally tested the intramolecular handover model using genetic and biochemical approaches. According to the AlphaFold-predicted structures, the conserved YY motif (Y53/Y54) of AAGAB is at the σ2:GD binding interface in the AAGAB:α:σ2 trimer (Fig. 5B). To examine the function of the YY motif in AP2 assembly, we introduced point mutations (Y53R/Y54R) into the YY motif and expressed the AAGAB mutant in *AAGAB* KO human cells. We observed that the AAGAB YY mutant failed to restore mature AP2 adaptors on the cell surface when expressed in *AAGAB* KO human cells (Fig. 6, A to C), confirming the critical role of the YY motif in AP2 assembly.

A prediction of the intramolecular handover model is that the YY mutations in the GD would impair AAGAB’s binding to not only σ2 but also α, due to the role of the YY motif in mediating the attachment of the α:σ2 hemicomplex to the GD of AAGAB. We observed that in human cells, the AAGAB YY mutant bound normally to α in the absence of σ2 (Fig. 6D), consistent with the role of the CTD in α binding (fig. S1, B to E). When both α and σ2 were coexpressed in human cells, neither bound to the AAGAB YY mutant (Fig. 6D). Similar findings were observed with the AAGAB DFEAV mutant, which carries mutations in additional residues at the GD:σ2 binding interface (D151R/F153R/E155R/A168R/V170R) in the G form of the AAGAB:α:σ2 heterotrimer (Fig. 5B). This mutant bound normally to α in human cells but lost the interaction when σ2 was present (fig. S8A). We next examined the effects of the YY mutations using recombinant proteins expressed in *E. coli* (fig. S8B). The AAGAB YY mutant bound normally to the α subunit in the absence of σ2 (fig. S8, C and D). However, when σ2 was coexpressed, the AAGAB YY mutant no longer interacted with either α or σ2, whereas WT AAGAB remained associated with both (fig. S8C). These results are fully consistent with the intramolecular handover model: When the α:σ2 hemicomplex dissociates from the CTD but cannot be received by the GD of the AAGAB YY mutant, the conformationally immature hemicomplex is released from the AAGAB molecule and is likely targeted for degradation by the cellular quality control system, thereby disrupting the AP2 assembly process.

Last, we directly examined whether the GD of AAGAB could displace the α:σ2 hemicomplex from the CTD. While efficient handover of the α:σ2 hemicomplex in the cell requires a direct linkage between the CTD and GD of AAGAB, the GD is expected to displace the α:σ2 hemicomplex from the CTD in vitro when added at a high concentration. To test this possibility, recombinant GD proteins were added to the AAGAB-CTD:α:σ2 trimeric complex isolated from *E. coli*. We observed that, in the presence of the GD, the α:σ2 was released from the CTD of AAGAB and transferred to the GD (Fig. 6, E and F), confirming a CTD-to-GD handover of the α:σ2 hemicomplex.

Together, these data demonstrate that the CTD and GD of AAGAB play distinct and complementary roles in AP2 assembly: The CTD initiates the formation of the α:σ2 hemicomplex, while the GD receives the hemicomplex from the CTD. It is only through this intramolecular handover mechanism that a conformationally mature α:σ2 hemicomplex can be formed, serving as a platform for the subsequent steps of AP2 assembly.

## DISCUSSION

**Fig. 7.**
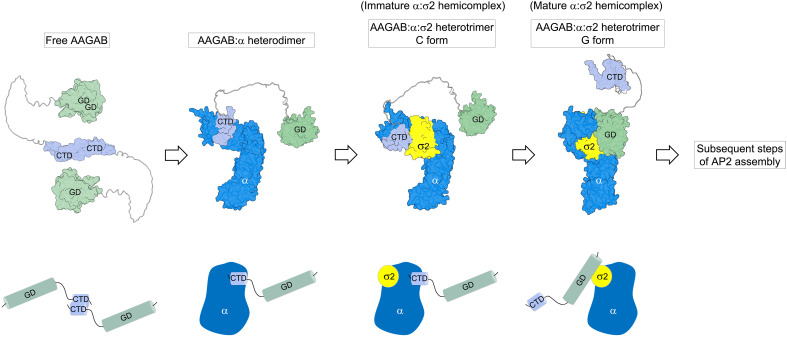
Model of the bi-handed chaperone AAGAB in regulating AP2 assembly. The diagram of free AAGAB, which forms a homodimer, is based on the crystal structure of AAGAB-CTD (PDB: 7TWD) (*26*). Structural models of the GD and LD domains are derived from the predicted structure of WT AAGAB (AlphaFold Protein Structure Database: AF-Q6PD74-F1). The remaining diagrams are based on experimentally validated structural models described in Figs. 4D and 5C. Cartoon representations are included below each structural model to illustrate protein binding interfaces more clearly. For clarity, only the core domain of the α subunit is shown.

In this work, we identified AAGAB as the founding member of a bi-handed assembly chaperone family and elucidated its molecular mechanism of action. AAGAB uses its CTD to recruit the α subunit of AP2, forming an AAGAB:α dimer (Fig. 7). Subsequently, σ2 joins the AAGAB:α dimer to assemble into an AAGAB:α:σ2 trimer (the C form), where an α:σ2 hemicomplex is formed and tethered to the CTD of AAGAB through a binding interface between α and the CTD of AAGAB. However, this initially formed α:σ2 hemicomplex remains conformationally immature. Next, the α:σ2 hemicomplex is transferred from the CTD to the GD of AAGAB, leading to its conformational maturation (Fig. 7). In the resulting AAGAB:α:σ2 trimer (the G form), the α:σ2 hemicomplex is attached to AAGAB through a binding interface between σ2 and the GD of AAGAB. Once the G form of the AAGAB:α:σ2 trimer is achieved, AAGAB completes its role in the AP2 assembly pathway—to assemble a conformationally mature α:σ2 hemicomplex. This α:σ2 hemicomplex then serves as a seed for the incorporation of the β2 and μ2 subunits, with the assistance of another assembly chaperone, CCDC32 (*40*).

The intramolecular handover mechanism of AAGAB involves a series of sequential molecular interactions with multiple intermediate stages, highlighting that although stabilizing two protein subunits and bringing them into proximity may be relatively straightforward, assembling them into a properly paired dimeric complex is a more challenging task. This intramolecular handover mechanism leverages the complementary functions of AAGAB’s two substrate-binding domains—its CTD and GD. Through a division of labor, these domains cooperatively construct a conformationally mature α:σ2 hemicomplex that is competent for subsequent steps in AP2 assembly. The CTD directly binds the α subunit, serving a dual role by recruiting α and initiating hemicomplex formation. The GD, through its direct interaction with σ2, receives the hemicomplex from the CTD and enables its conformational maturation. Because the AAGAB:σ2 dimer represents a dead-end configuration incapable of recruiting α (Fig. 4), stable binding of σ2 to AAGAB is prevented in cells, likely via quality-control mechanisms. Whether the GD transiently interacts with σ2 to bring it near the AAGAB:α dimer remains an open question. Unlike folding chaperones, assembly chaperones like AAGAB operate without energy input, relying solely on the free energy of protein-protein interactions. Together, these findings support the idea that protein complexes are evolutionarily selected to assemble through ordered and mechanistically regulated pathways (*47*).

The C form of the AAGAB-CTD:α:σ2 trimer is an intermediate structure upstream of the G form but is biochemically stable in *E. coli* and in vitro (Figs. 4F and 6E). Thus, the α:σ2 hemicomplex does not spontaneously dissociate from the CTD of AAGAB, suggesting that its release is actively triggered by the GD. Since the α:σ2 hemicomplex undergoes a conformational change upon release from the CTD of AAGAB (Fig. 5), its rebinding to the CTD is prevented, thereby ensuring directionality in the assembly reaction.

Our findings also provide molecular insights into AAGAB-linked pathogenesis. Heterozygous mutations in AAGAB cause punctate palmoplantar keratoderma type 1, a skin disorder, and are associated with an increased incidence of cancer (*48*–*50*). Many of the disease-causing mutations result in truncated AAGAB proteins lacking the CTD (*26*, *49*–*52*). Data presented in this work indicate that these mutant proteins are defective at the initiation stage of the AP2 assembly pathway—the recruitment and pairing of α and σ2 into a hemicomplex.

Our findings raise several key questions. First, how does AAGAB regulate the assembly of other adaptor protein (AP) complexes? In addition to AP2, AAGAB assists the assembly of AP1 and AP4 (*26*, *27*, *53*), which are involved in protein transport at the Golgi and endosomes (*2*, *14*, *54*). The AAGAB-assisted AP assembly is part of a biological pathway known as chaperone-assisted AP assembly (*17*, *26*, *27*, *40*). Since AP1 and AP4 adopt a configuration similar to AP2 and are also depleted in AAGAB-deficient cells (*27*, *53*), their assembly likely involves an intramolecular handover mechanism as well. Nevertheless, the specific assembly routes for these APs might differ. For example, a dimeric hemicomplex of AP1 or AP4 could initially form at the GD of AAGAB before being transferred to the CTD, meeting the unique structural demands of each AP. Another open question is how AP2 assembly progresses after AAGAB builds the α:σ2 hemicomplex. Specifically, it would be important to determine how the α:σ2 hemicomplex is transferred from AAGAB to CCDC32 and how μ2 and β2 are subsequently incorporated. Last, how do bi-handed assembly chaperones regulate the formation of other protein complexes? We anticipate that this important family of chaperones will continue to expand as our understanding of protein complex assembly deepens. Since protein complexes encounter similar challenges when forming dimeric intermediates, bi-handed assembly chaperones are expected to play broad roles in protein complex assembly, and the intramolecular handover mechanism is likely conserved across this family of chaperones.

## MATERIALS AND METHODS

### Mammalian gene expression plasmids

Mammalian expression plasmids encoding WT AAGAB and AP2 subunits were generated previously (*17*). AAGAB LD deletion mutants were created using a site-directed mutagenesis kit (Agilent, catalog no. R1018). A DNA fragment encoding the flipped AAGAB variant (amino acids 258 to 315, 188 to 247, and 1 to 187) was synthesized by Integrated DNA Technologies (IDT) and subcloned into the Hind III and Xba I sites of the p3xFLAG-7.1 vector (Sigma-Aldrich, catalog no. E7533). To generate 3xFLAG-mCherry-CTD, DNA fragments encoding mCherry (digested with Hind III and Eco RI) and the AAGAB CTD (amino acids 258 to 315, digested with Eco RI and Xba I) were subcloned into the Hind III and Xba I sites of the p3xFLAG-7.1 vector. AAGAB point mutations were introduced using site-directed mutagenesis.

### Mammalian cell culture and gene KO

Human embryonic kidney (HEK) 293T cells and HeLa cells were grown in Dulbecco’s modified Eagle’s medium supplemented with 10% FB Essence (VWR, #10803-034) and penicillin/streptomycin (Thermo Fisher Scientific, #15140122). The cell lines were maintained in a humidified 37°C incubator with 5% CO_2_ and were routinely tested for mycoplasma.

To delete the *AAGAB* gene, two guide RNA (gRNA) sequences targeting early constitutive exons of the gene were selected: 5′-CAGCTGGTCTCCTGAGAAGA-3′ and 5′-GCAGTAACAAGAAATTTGTT-3′. One of the gRNAs was subcloned into the pLenti-CRISPR-V2 vector (Addgene, #52961). The second gRNA was subcloned into a modified version of the pLentiGuide-Puro vector (Addgene, #52963), in which the puromycin selection marker was substituted with a hygromycin selection marker. CRISPR plasmids were transiently transfected into HEK 293T cells together with pAdVAntage (Promega, #E1711), pCMV-VSV-G (Addgene, #8454), and psPAX2 (Addgene, #12260) using a previously established procedure (*17*). HEK 293T culture media containing lentiviral particles were collected over four rounds and centrifuged in a Beckman SW28 rotor at 25,000 rpm (113,000*g*) for 1.5 hours at 4°C. Lentiviral pellets were resuspended in phosphate-buffered saline (PBS) buffer and used to infect HeLa cells. Infected HeLa cells were sequentially selected using puromycin (1 μg/ml; Millipore-Sigma, #3101118) and hygromycin B (500 μg/ml; Thermo Fisher Scientific, #10687010). Loss of AAGAB expression in the pooled KO cells was confirmed using immunoblotting.

### Flow cytometry

Cells grown on tissue culture plates were rinsed three times with KRH buffer [12 mM Hepes (pH 7.0), 121 mM NaCl, 4.9 mM KCl, 1.2 mM MgSO_4_, and 0.33 mM CaCl_2_] and then blocked with 5% fetal bovine serum (FBS) in KRH buffer at 4°C. To quantify surface TfR levels, unpermeabilized HeLa cells were stained using allophycocyanin (APC)–conjugated anti-TfR (BioLegend, #334108). After dissociating from the plates using Accutase, APC fluorescence was measured on a CyAn ADP Analyzer (Beckman Coulter). Mean APC fluorescence of mutant HeLa cells was normalized to that of WT cells. Data from populations of approximately 5000 cells were analyzed using FlowJo software, version 10 (FlowJo LLC), based on experiments conducted in biological triplicates.

### Immunofluorescence

Cells grown on coverslips were fixed using 4% paraformaldehyde and permeabilized in PBS containing 5% FBS and 0.2% saponin. The endogenous AP2 α subunit was labeled using anti–α-adaptin antibodies (Santa Cruz Biotechnology, #sc-17771, RRID: AB_2274034) and Alexa Fluor 568–conjugated secondary antibodies (Thermo Fisher Scientific, #A11004, RRID: AB_2534072) or Alexa Fluor 488–conjugated secondary antibodies (Thermo Fisher Scientific, #A11008, RRID: AB_142165). The cell surface was stained with CF405-conjugated concanavalin A (Biotum, #29074). Confocal images were captured using a 100× oil immersion objective on a Yokogawa/Olympus CV1000 spinning disk confocal microscope. Images were processed using Fiji software.

### Immunoblotting and immunoprecipitation

To prepare whole cell lysates for immunoblotting, cells grown in 24-well plates were lysed in SDS protein sample buffer [80 mM tris (pH 6.8), 2% SDS, 10% glycerol, 0.0006% bromophenol blue, and 0.1 M dithiothreitol (DTT)]. Proteins in the cell lysates were resolved on 8% bis-tris SDS–polyacrylamide gel electrophoresis (SDS-PAGE), transferred to polyvinylidene difluoride (PVDF) membranes, and stained using unlabeled primary antibodies and horseradish peroxidase (HRP)–conjugated secondary antibodies. Primary antibodies used in immunoblotting included polyclonal anti-AAGAB antibodies (Bethyl Laboratories, #A305-593A, RRID: AB_2782752), monoclonal anti–AP2-α antibodies (BD Biosciences, #610502, RRID: AB_397868), polyclonal anti–AP2-β antibodies (Bethyl Laboratories, #A304-719A, RRID: AB_2620914), monoclonal anti–AP2-μ antibodies (BD Biosciences, #611350), and polyclonal anti–AP2-σ antibodies (Abcam, #ab128950, RRID: AB_11140842). Secondary antibodies used in immunoblotting included HRP-conjugated anti-rabbit antibodies (Sigma-Aldrich, #A6154, RRID: AB_258284) and HRP-conjugated anti-mouse antibodies (Sigma-Aldrich, #A6782, RRID: AB_258315). FLAG-tagged proteins were directly detected using HRP-conjugated anti–FLAG M2 antibodies (Sigma-Aldrich, #A8592, RRID: AB_439702). Hemagglutinin (HA)–tagged proteins were directly detected using HRP-conjugated anti-HA antibodies (Roche, #12013819001, RRID: AB_390917).

In immunoprecipitation experiments, cells were lysed in IP buffer [25 mM Hepes (pH 7.4), 150 mM NaCl, 10 mM Na_3_PO_4_, 2.7 mM KCl, 0.5% CHAPS, 1 mM DTT, and a protease inhibitor cocktail]. After centrifugation to remove cell debris, proteins were immunoprecipitated using anti–FLAG M2 antibodies and protein A/G agarose beads. Immunoprecipitated proteins were resolved on 8% bis-tris SDS-PAGE and detected using immunoblotting. Intensities of protein bands on immunoblots were quantified using Fiji.

### Recombinant protein expression and pull-down assays

Plasmids expressing His_6_-SUMO–tagged WT AAGAB, glutathione *S*-transferase (GST)–tagged AP2 α trunk domain (amino acids 1 to 621), and untagged WT σ2 were generated in a previous study (*17*). His_6_-SUMO–tagged AAGAB GD (amino acids 1 to 177) and CTD (amino acids 258 to 315) were generated in a similar manner as the WT protein. Recombinant proteins were expressed in BL21 (DE3) *E. coli* (Stratagene, #230132). When the OD_600_ (optical density at 600 nm) of *E. coli* cultured in 2’YT media reached approximately 0.6, 1 mM isopropyl-β-d-thiogalactopyranoside (GoldBio, #I2481C100) was added to induce protein expression. After 3 hours of incubation at 37°C, the bacterial cells were harvested and lysed. Following centrifugation to remove insoluble fractions, recombinant proteins were isolated using glutathione beads (Thermo Fisher Scientific, #16101) or nickel beads (Thermo Fisher Scientific, #88222) as previously described (*17*, *26*).

In the CTD displacement assay, *E. coli* lysates expressing the GD of AAGAB were added to the GST-α:σ2:AAGAB-CTD complex anchored to glutathione beads. After incubation at 4°C for 1 hour, the beads were washed with the cell lysis buffer supplemented with 60 mM imidazole. Proteins were resolved on 8% bis-tris SDS-PAGE and detected either by Coomassie blue staining or, after transfer to PVDF membranes, by a reversible protein staining solution (Thermo Fisher Scientific, #YC369027) or by immunoblotting.

### Structural prediction and analysis

The structures of AAGAB:AP2 complexes were predicted using AlphaFold3 and AlphaFold2 multimer (implemented in the ColabFold interface) with default settings (*55*, *56*). Ten independent structural models were generated for each protein complex, and the quality of the predicted models was assessed through their ipTM scores, predicted template modeling scores, predicted alignment error (PAE) plots, and pLDDT scores (*55*, *56*). A structural model for a protein complex was selected on the basis of the scores above and, whenever possible, its correlation with experimental data. PAE plots were generated using the PAE Viewer (*42*). Structural analysis was conducted using UCSF ChimeraX (v1.7) (*42*) and PDBePISA (*57*). The chains of the AlphaFold-predicted models were aligned to the fully assembled AP2 adaptor (PDB: 4UQI) (*34*) using UCSF ChimeraX’s Matchmaker tool with default parameters (*58*). To evaluate structural variations, RMSD scores for Cα atoms were calculated between two structures via Matchmaker pairwise sequence alignment. The RMSD values were visualized in ChimeraX. The AlphaFold-predicted structural models shown in this work are listed in table S1.

## Supplementary Materials

### The PDF file includes:

Figs. S1 to S8 Table S1 Legends for datasets S1 to S5

### Other Supplementary Material for this manuscript includes the following:

Datasets S1 to S5
