# Supplementary material for: Bi-handed assembly chaperones regulate protein complex assembly through an intramolecular handover mechanism
Source: Sci Adv. 2025 Sep 12;11(37):eadw9158. doi: 10.1126/sciadv.adw9158 (PMC12429027; doi:10.1126/sciadv.adw9158)
Supplement: Supplementary file 1 — Figs. S1 to S8 Table S1 Legends for datasets S1 to S5 [file sciadv.adw9158_sm.pdf]

Science Advances
AAAS

Supplementary Materials for

Bi-handed assembly chaperones regulate protein complex assembly through an intramolecular handover mechanism

Jingyi Wu *et al.*

Corresponding author: Qian Yin, yin@bio.fsu.edu; Jingshi Shen, jingshi.shen@colorado.edu

Sci. Adv. **11**, eadw9158 (2025)
DOI: 10.1126/sciadv.adw9158

The PDF file includes:

Figs. S1 to S8
Table S1
Legends for datasets S1 to S5

Other Supplementary Material for this manuscript includes the following:

Datasets S1 to S5

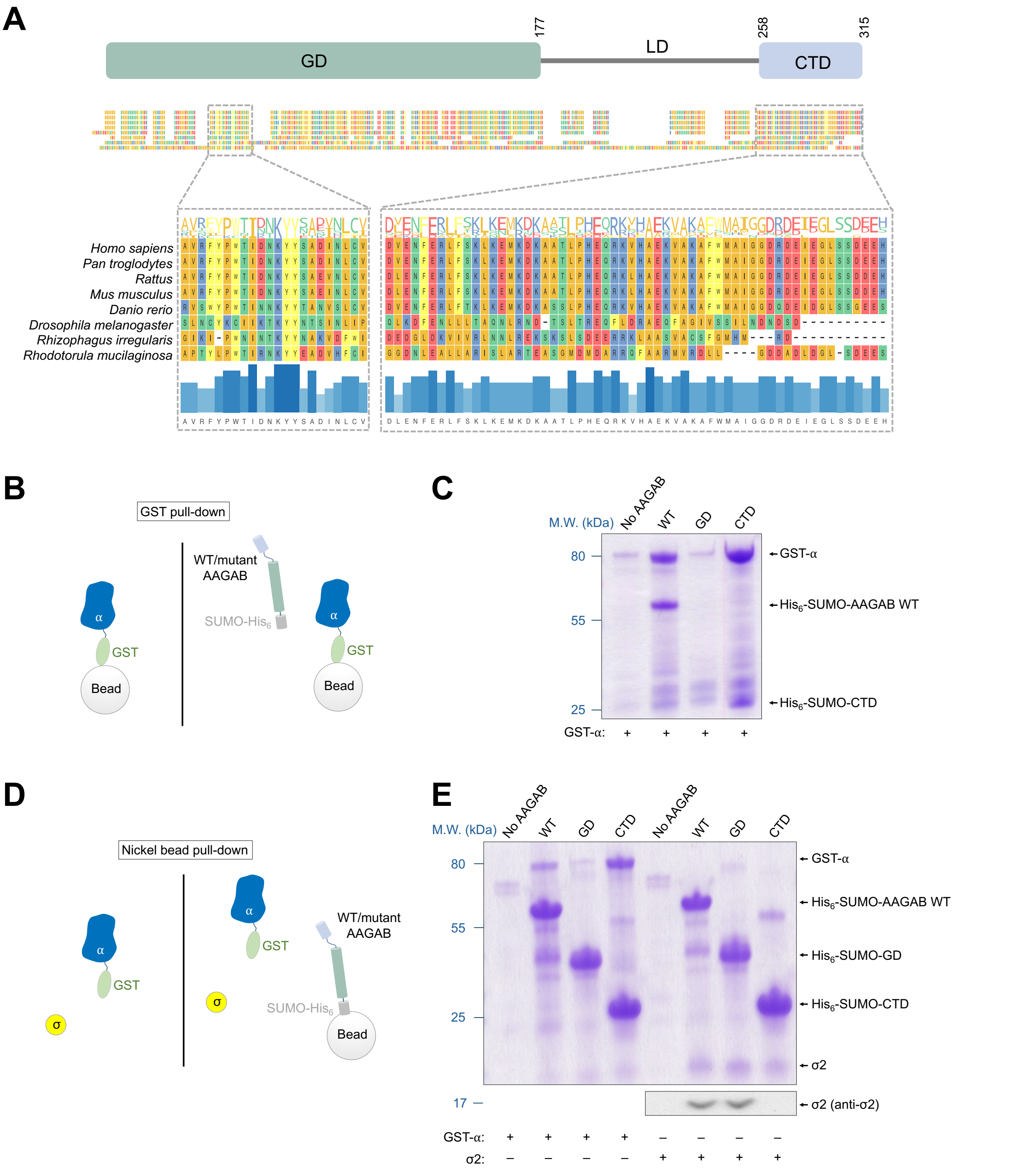

Figure S1. The GD and CTD of AAGAB directly bind and stabilize the σ2 and α subunits of the AP2 adaptor. (A) Diagram of the human AAGAB protein (top) and alignment of AAGAB protein sequences from representative species (bottom). Protein sequences were aligned using MUSCLE, with amino acids colored according to their side-chain chemistry. Protein sequence logos and consensus bars are shown. **(B)** Diagram of GST pull-down assays detecting the interaction of AAGAB with the AP2 α subunit. His_6-SUMO-tagged AAGAB (WT or mutants)

was co-expressed with GST-tagged α (trunk domain, a.a. 1-621) in *E. coli*. Lysates of *E. coli* expressing the indicated proteins were used for pull-down assays. **(C)** Representative Coomassie blue-stained gel showing the binding of GST-tagged α to His_6-SUMO-tagged AAGAB, as depicted in B. **(D)** Diagram of a nickel bead pull-down assay detecting the interaction of AAGAB with σ2 and α. His_6-SUMO-tagged AAGAB (WT or mutants) was co-expressed with GST-tagged α (trunk domain) or untagged WT σ2 in *E. coli*. Lysates of *E. coli* expressing the indicated proteins were used for pull-down assays. **(E)** Representative Coomassie blue-stained gel (top) and immunoblot (bottom) showing the binding of GST-tagged α and untagged σ2 to His_6-SUMO-tagged AAGAB, as depicted in D. Related to Figure 1.

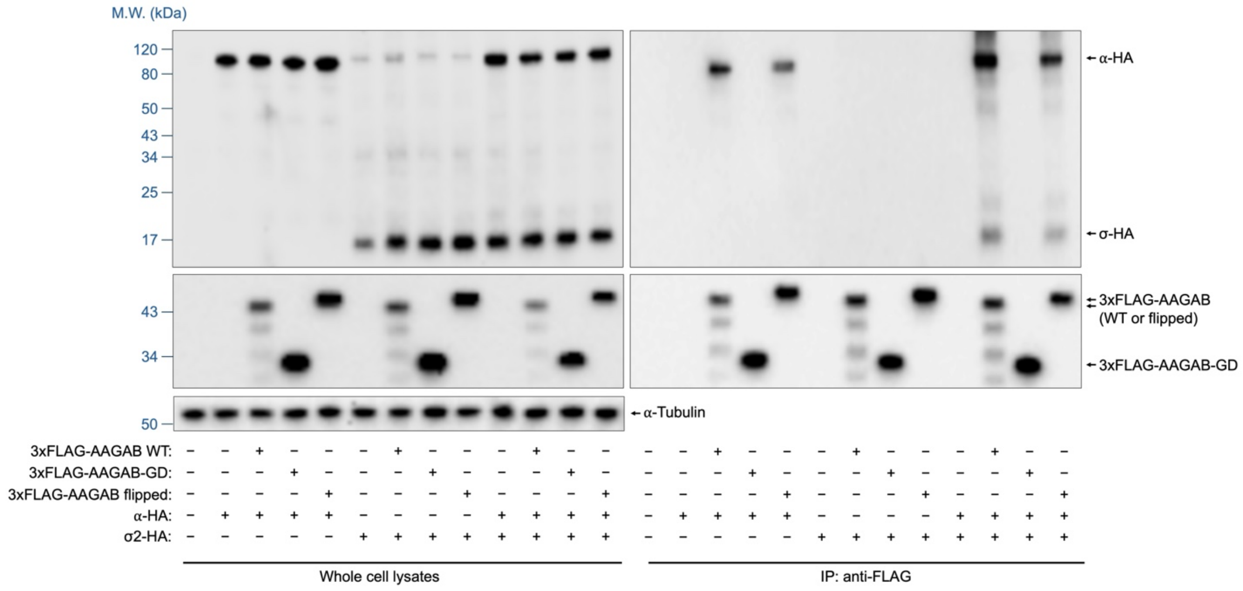

Figure S2. Interactions of α and σ2 with a flipped AAGAB variant and the GD of AAGAB. Co-IP experiments were performed as described in Fig. 4C. Representative immunoblots show the interactions of HA-tagged α and σ2 with 3xFLAG-tagged WT AAGAB, a flipped AAGAB variant, and the GD of AAGAB in HeLa cells. Related to Figures 3 and 4.

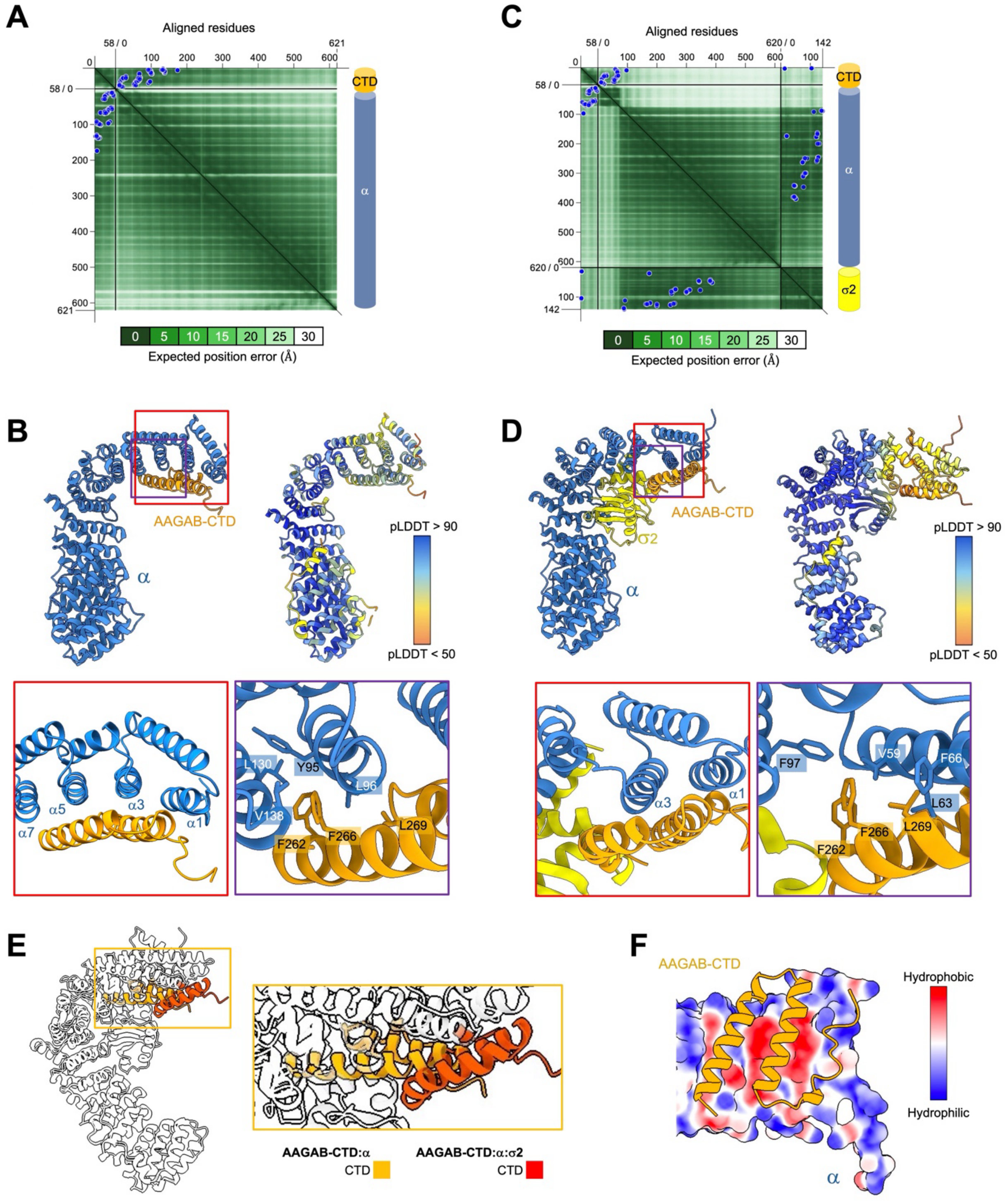

Figure S3. Structural models of the AAGAB-CTD:α dimer and AAGAB-CTD:α:σ2 trimer. **(A)** PAE heatmap of the AlphaFold-predicted structure of the AAGAB-CTD:α dimer shown in Fig. 4D (left). Interchain interactions are represented as blue dots. **(B)** Top: AlphaFold-predicted structure of the AAGAB-CTD:α dimer, shown with coloring by protein subunits (left) and by pLDDT scores (right). Bottom: enlarged view of the binding interface between the CTD of AAGAB and the α subunit within the AAGAB-CTD:α dimer. **(C)** PAE heatmap of the AlphaFold-

predicted structure of the AAGAB-CTD:α:σ2 trimer shown in Fig. 4D (right). **(D)** Top: AlphaFold-predicted structures of the AAGAB-CTD:α:σ2 trimer, shown with coloring by protein subunits (left) and by pLDDT scores (right). Bottom: enlarged view of the binding interface between the CTD of AAGAB and the α subunit within the AAGAB-CTD:α:σ2 trimer. **(E)** Superposition of the AlphaFold-predicted structures of the AAGAB-CTD:α dimer and the AAGAB-CTD:α:σ2 trimer. The inset shows the binding interface between α and the CTD of AAGAB. **(F)** Structural model depicting the hydrophobic regions of the AP2 α subunit shielded by the CTD of AAGAB. Related to Figure 4.

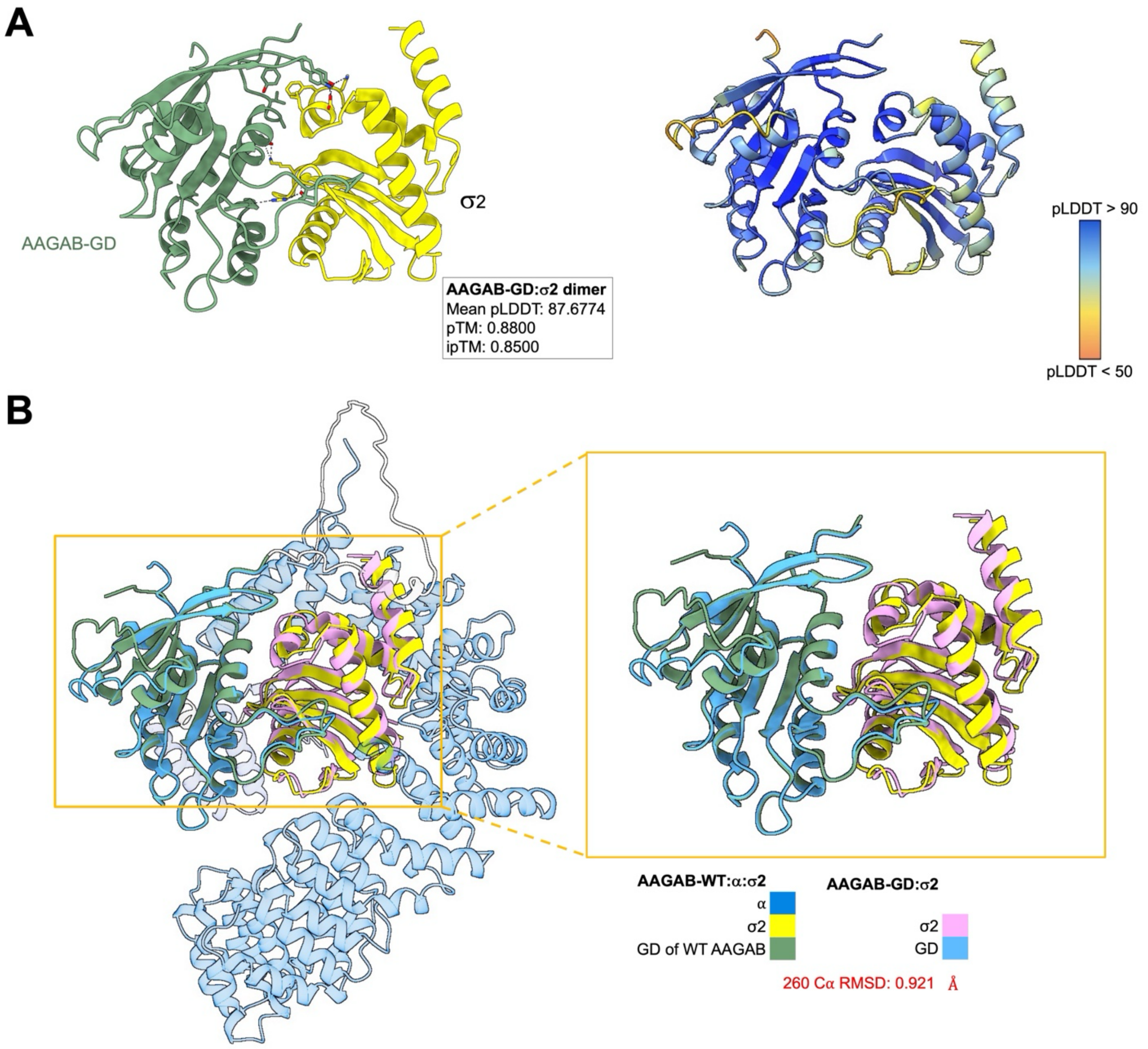

Figure S4. Structural model of the AAGAB-GD:σ2 dimer. (A) AlphaFold-predicted structure of the AAGAB-GD:σ2 dimer, shown with coloring by protein subunits (left) and by pLDDT scores (right). The prediction was performed using the GD (amino acids 1-177) of AAGAB and WT σ2. The PDB/CIF file of the predicted structure is included in Supplementary Dataset 2. **(B)** Superposition of the AlphaFold-predicted structures of the AAGAB-GD:σ2 dimer and the AAGAB-WT:α:σ2 trimer (described in Fig. 5A-B). The inset shows the binding interface between σ2 and the GD of AAGAB. The Root Mean Square Deviation (RMSD) scores, which measure similarity between two structures, were calculated using UCSF ChimeraX. The RMSD score between 260 pruned atom pairs is 0.921 Å (2.427 Å across all 319 pairs). Related to Figure 4.

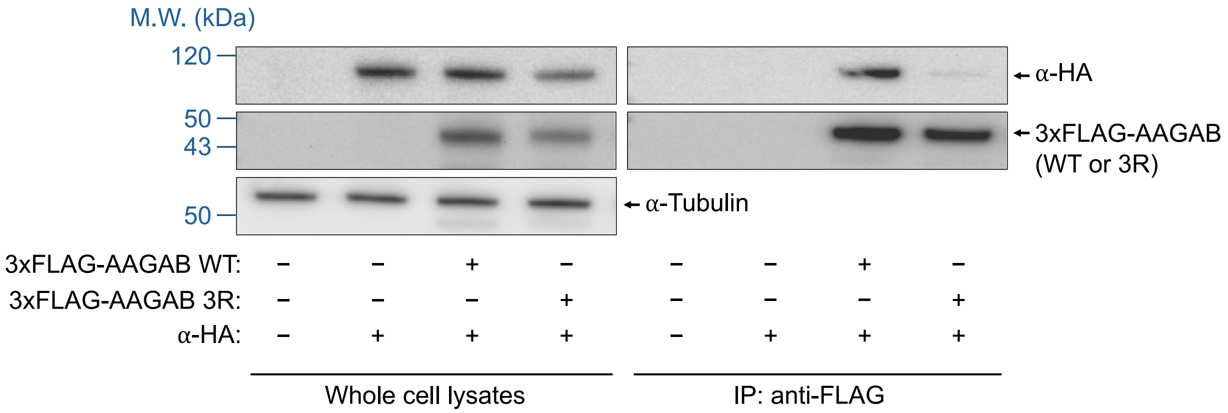

Figure S5. Mutations in the CTD of AAGAB impair its binding to the α subunit in human cells. Co-IP was performed as described in Fig. 4C. Representative immunoblots show the interactions of HA-tagged α and σ2 with 3xFLAG-tagged WT AAGAB or the 3R mutant (F262R, F266R, and L269R) in HeLa cells.

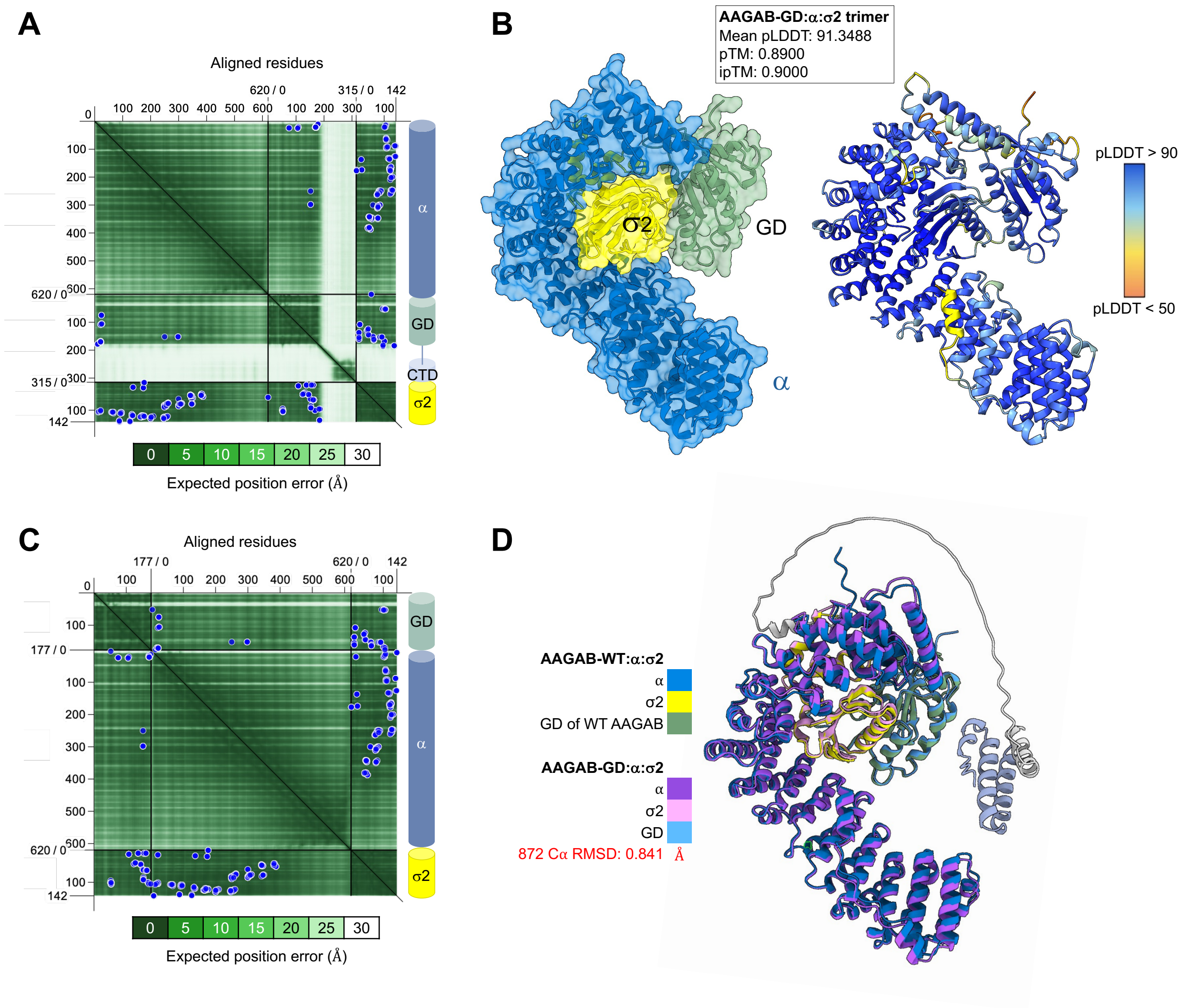

Figure S6. Structural models of the AAGAB-WT:α:σ2 and AAGAB-GD:α:σ2 trimers. (A) PAE heatmap of the AlphaFold-predicted structure of the AAGAB-WT:α:σ2 trimer shown in Fig. 5A. Interchain interactions are represented as blue dots. **(B)** AlphaFold-predicted structure of the AAGAB-GD:α:σ2 trimer, shown with coloring by protein subunits (left) and by pLDDT scores (right). The prediction was performed using the GD (amino acids 1-177) of AAGAB, the α trunk domain, and WT σ2. The PDB/CIF file of the predicted structure is included in Supplementary Dataset 5. **(C)** PAE heatmap of the structure of the AAGAB-GD:α:σ2 trimer shown in (b). **(D)** Superposition of the AlphaFold-predicted structures of the AAGAB-WT:α:σ2 and AAGAB-GD:α:σ2 trimers. The RMSD between 872 pruned atom pairs is 0.841 Å (1.863 Å across all 939 pairs). Related to Figure 5.

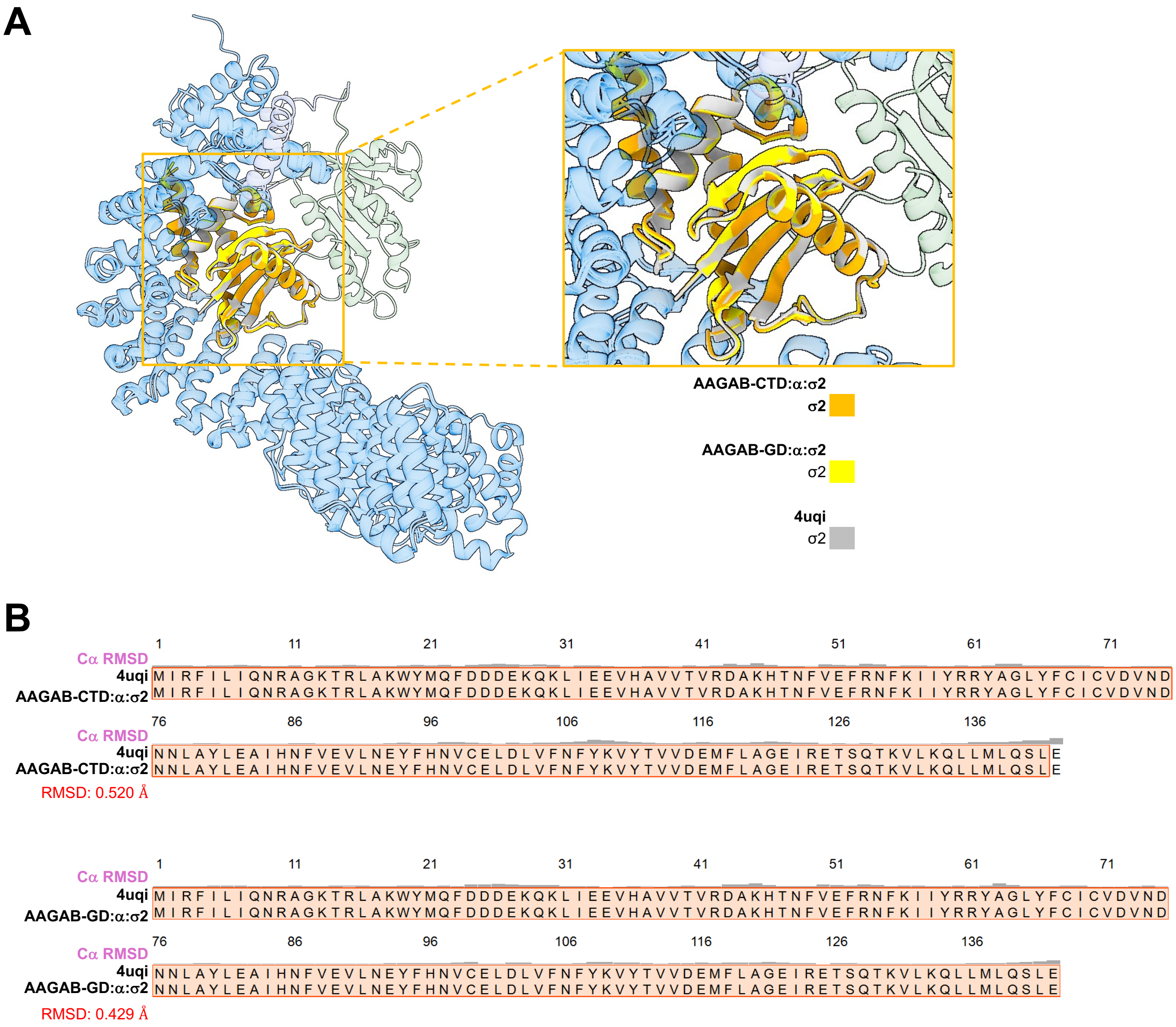

Figure S7. Similar Structures of σ2 in the full AP2 adaptor and AAGAB-AP2 complexes. (A) Superposition of the structures of AAGAB-CTD:α:σ2, AAGAB-GD:α:σ2, and the full AP2 adaptor. The inset shows an enlarged view of σ2. The full AP2 adaptor structure is based on a crystal structure (PDB: 4UQI), while the other complexes are based on AlphaFold predictions described in Figs. 4D and S4B. The color schemes for AAGAB and α are consistent with those in Fig. 5. For clarity, the β2 and μ2 subunits are not shown. **(B)** Structural comparison of σ2 between the indicated protein complexes. Related to Figure 5.

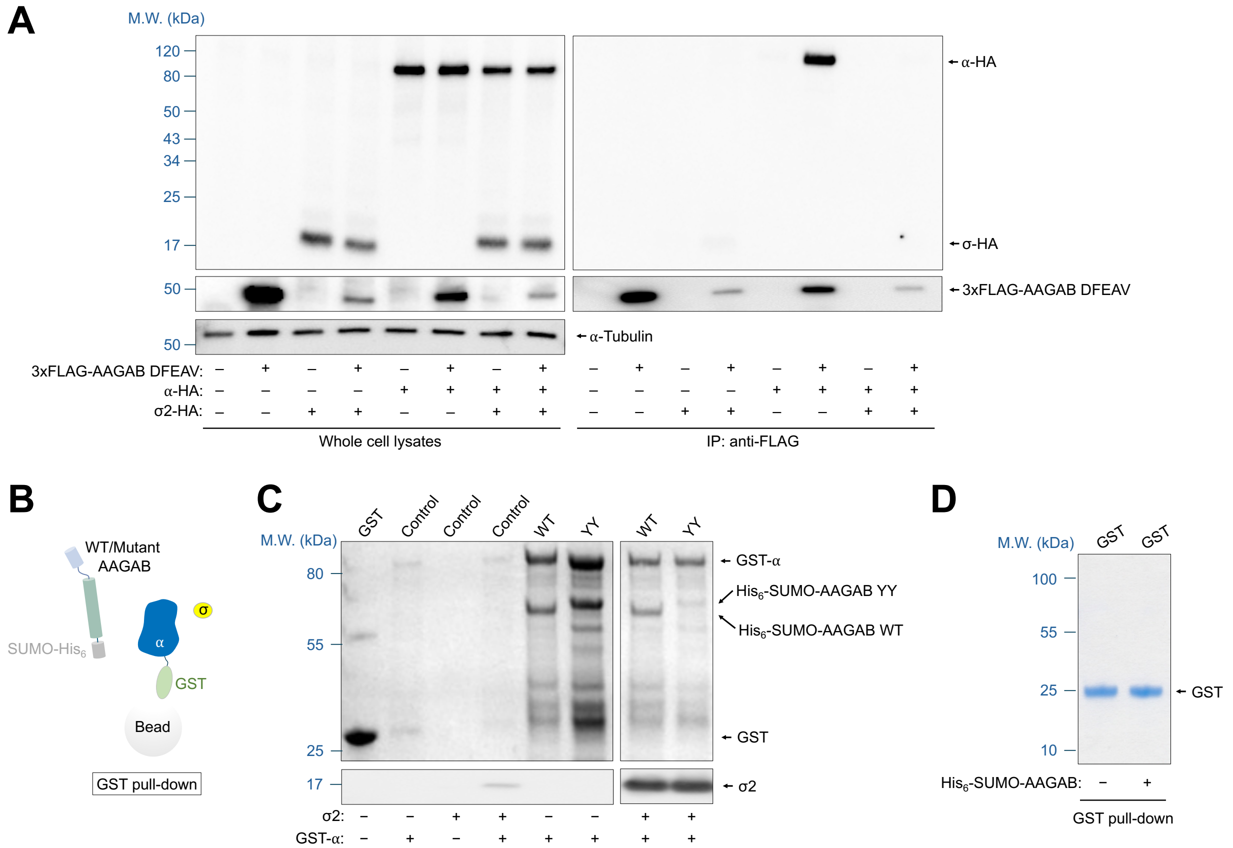

Figure S8. Mutations at the AAGAB-GD:σ2 binding interface impair the intramolecular handover of the α:σ2 hemicomplex. (A) Co-IP experiments were performed as described in Fig. 4C. Representative immunoblots show the interaction of HA-tagged α and σ2 with the 3xFLAG-tagged AAGAB DFEAV mutant (D151R/F153R/E155R/A168R/V170R) in HeLa cells. **(B)** Diagram of a GST pull-down assay used to assess the binding of AAGAB (WT or YY mutant) to α and σ2. His_6-SUMO-tagged AAGAB was co-expressed with GST-tagged α (trunk domain) and untagged WT σ2 in *E. coli*. Lysates from *E. coli* expressing the indicated proteins were used for GST pull-down assays. **(C)** Representative PVDF membranes showing the binding of AAGAB (WT or YY mutant) to GST-tagged α and untagged σ2. Proteins from GST pull-down assays were resolved by SDS-PAGE, transferred to PVDF membranes, and stained with a protein staining solution. **(D)** Representative Coomassie blue-stained gel demonstrating that GST alone does not bind to AAGAB. Protein expression and GST pull-down were performed as described in B-C. Related to Figure 6.

Table S1. List of AlphaFold-predicted structures

AAGAB-AP2 complex	Input	AlphaFold	ipTM	pTM	pLDDT	Figure	PDB/CIF file
AAGAB-CTD:α dimer	a.a. 258-315 of human AAGAB and a.a. 1-621 of human AP2 α	AlphaFold3	0.83	0.76	85.0976	Fig. 4	Supplementary Dataset 1
AAGAB-GD:σ2 dimer	a.a. 1-177 of human AAGAB and a.a. 1-621 of human AP2 α	AlphaFold3	0.85	0.88	87.6774	Fig. S3	Supplementary Dataset 2
AAGAB-CTD:α:σ2 trimer	a.a. 258-315 of human AAGAB, a.a. 1-621 of human AP2 α, and WT human AP2 σ	AlphaFold2	0.75	0.80	84.7394	Fig. 4	Supplementary Dataset 3
AAGAB-WT:α:σ2 trimer	WT human AAGAB, a.a. 1-621 of human AP2 α, and WT human AP2 σ	AlphaFold2	0.77	0.79	83.0574	Fig. 5	Supplementary Dataset 4
AAGAB-GD:α:σ2 trimer	a.a. 1-177 of human AAGAB, a.a. 1-621 of human AP2 α, and WT human AP2 σ	AlphaFold2	0.9	0.89	91.3488	Fig. S4	Supplementary Dataset 5

Additional Supplementary Files:

Supplementary Dataset 1. An AlphaFold-predicted structure of the AAGAB-CTD:α dimer (CIF file).

Supplementary Dataset 2. An AlphaFold-predicted structure of the AAGAB-GD:σ2 dimer (CIF file).

Supplementary Dataset 3. An AlphaFold-predicted structure of the AAGAB-CTD:α:σ2 trimer (CIF file).

Supplementary Dataset 4. An AlphaFold-predicted structure of the AAGAB-WT:α:σ2 trimer (PDB file).

Supplementary Dataset 5. An AlphaFold-predicted structure of the AAGAB-GD:α:σ2 trimer (PDB file).
